# Efficacy of SCF drug conjugate targeting c-KIT in gastrointestinal stromal tumor

**DOI:** 10.1186/s12916-022-02465-3

**Published:** 2022-08-24

**Authors:** Dengyang Zhang, Chunxiao He, Yao Guo, Jianfeng Li, Bo Li, Yuming Zhao, Liuting Yu, Zhiguang Chang, Hanzhong Pei, Ming Yang, Na Li, Qi Zhang, Yulong He, Yihang Pan, Zhizhuang Joe Zhao, Changhua Zhang, Yun Chen

**Affiliations:** 1 Edmond H. Fischer Translational Medical Research Laboratory, Scientific Research Center, The Seventh Affiliated Hospital, Sun Yat-Sen University, Shenzhen, 518107 Guangdong China; 2 Digestive Diseases Center, The Seventh Affiliated Hospital, Sun Yat-Sen University, Shenzhen, 518107 Guangdong China; 3 Department of Pathology, University of Oklahoma Health Sciences Center, Oklahoma City, OK 73104 USA

**Keywords:** GIST, SCF, DM1, Targeted therapy

## Abstract

**Background:**

Gastrointestinal stromal tumor (GIST) is a rare type of cancer that occurs in the gastrointestinal tract. The majority of GIST cases carry oncogenic forms of KIT, the receptor for stem cell factor (SCF). Small molecule kinase inhibitor imatinib is effective in prolonging the survival of GIST patients by targeting KIT. However, drug resistance often develops during the therapeutic treatment. Here, we produced a SCF-emtansine drug conjugate (SCF-DM1) with favorable drug efficacy towards GIST cells.

**Methods:**

Recombinant human SCF (rhSCF) was expressed in *E. coli* cells and further purified with Ni–NTA Sepharose and Phenyl Sepharose. It was then conjugated with DM1, and the conjugated product SCF-DM1 was evaluated using in vitro cell-based assays and in vivo xenograft mouse model.

**Results:**

SCF-DM1 was effective in inhibiting imatinib-sensitive and -resistant GIST cell lines and primary tumor cells, with IC_50_ values of < 30 nM. It induced apoptosis and cell cycle arrest in GIST cells. In xenograft mouse model, SCF-DM1 showed favorable efficacy and safety profiles.

**Conclusions:**

rhSCF is a convenient and effective vector for drug delivery to KIT positive GIST cells. SCF-DM1 is an effective drug candidate to treat imatinib-sensitive and -resistant GIST.

**Supplementary Information:**

The online version contains supplementary material available at 10.1186/s12916-022-02465-3.

## Background

Gastrointestinal stromal tumor (GIST) is a type of soft tissue tumor that mainly occurs along the gastrointestinal (GI) tract. It originates from the interstitial cells of Cajal which lines in the wall of GI tract [1] and extends towards mucosa or/and serosa [2]. In the clinical practice, GIST can recur years after initial tumor excision, and demonstrate ability of distant metastasis. It metastasizes principally to liver and peritoneal surfaces, rarely to lymph nodes, lung and bone [3]. As the majority of GIST have relatively low mitotic rate, grading of cancer stage is only determined by mitotic count on an area that shows the highest level of mitotic activity [4]. The incidence of GIST is 0.70 per 100,000 people per year in the USA based on data from 2001 to 2015 [5]. The 5-year relative survival rate for GIST for all stages combined is 83% [6].

Gain-of-function mutated forms of KIT and PDGFR-alpha are two well-known oncogenic proteins which play pivotal role in the development and maintenance of GIST [1, 7]. KIT and PDGFR-alpha belong to the type III receptor tyrosine kinase family, members of which can lead to the occurrence of several cancers [8, 9]. Eighty-five percent of GIST contains either the mutant KIT or mutant PDGFR-alpha [10]. KIT mutation is the main oncogenic driver of GIST and can be found in 80% of the total GIST [11]. Activating mutations of KIT occur at the extracellular D5 domain, juxtamembrane domain (JM), tyrosine kinase 1 (TK1), and activation loop (A-loop) [12]. Gain-of-function mutations of KIT lead to constant phosphorylation and activation of its cytoplasmic tyrosine kinase domain. Auto-phosphorylated KIT constantly activates downstream PI3K, MAPK, PLC, and JAK signaling pathways, leading to the survival and expansion of GIST cells [12].

Therefore, KIT is a reasonable target for therapy of GIST. Imatinib is the FDA-approved first-line small molecule kinase inhibitor for GIST with mutant KIT, and it effectively inhibits the activation of D5-mutated and JM-mutated KIT in GIST patients [13, 14]. About 80% of advanced GIST patients can reach disease control (complete or partial response or stable disease) after imatinib therapy (400 mg/day) [12]. During imatinib therapy, drug resistance builds because of secondary mutations of KIT which confer imatinib ineffective. Also, imatinib is ineffective towards the GIST patients with wild-type KIT [15].

Novel therapeutic methods targeting KIT are in development in recent years. Antibody–drug conjugate (ADC) LOP608 shows good efficacy and safety profile in preclinical trial [16]. ADC NN2101-DM1 is effective to KIT-expressing cancer in xenograft mouse model [17]. ADC utilizes the specificity of antibody towards its target and the high toxicity of its conjugated drug. Emtansine (DM1) is one of the conjugated drugs, which induces apoptosis in cancer cells by inhibiting mitosis, but the high toxicity of DM1 restricts its clinical use as a single chemotherapeutic agent [18, 19]. As the concept of targeted therapy develops, DM1 achieves successes by conjugating to therapeutic antibodies [19, 20]. A prominent example is FDA-approved drug ado-trastuzumab emtansine that is used as a second-line medicine to treat HER2-positive metastatic breast cancer [21]. Cytokines are the natural, specific ligands for receptors, which could also be utilized to develop targeted drugs [22]. Ontak, a recombinant fusion protein of diphtheria toxin and IL-2, is a FDA-approved medicine used for treatment of CD25-positive cutaneous T cell lymphoma by targeting membrane IL-2 receptor [23].

Stem cell factor (SCF) is the natural ligand of KIT which exists in either soluble or membrane bound form due to whether containing the primary proteolytic-cleavage site encoded by exon 6 [24]. Soluble SCF forms non-covalently associated homodimer, which is needed for binding with KIT, inducing receptor homodimerization, structure rearrangement, and autophosphorylation of TK1. In normal cells, SCF induces KIT internalization. The internalized KIT is either recycled to membrane or ubiquitylated for lysosomal degradation, demonstrating a tight regulation of SCF/KIT signaling [25]. In mutant-KIT-driven cancers, mutant KIT is auto-phosphorylated and this autophosphorylation process is independent of SCF stimulation. As mutations of KIT do not occur at the binding interface of KIT [26], SCF can still bind with mutant KIT thereby inducing its internalization and decreasing its protein levels.

Mutant KIT retains the ability of binding with SCF, and therefore, SCF can be used as a reasonable vector to deliver cytotoxic drug(s) into GIST cancer cells. Also, the affinity (K_D_ value, 4 nM [27] or 570 pM [28]) between SCF and KIT is acceptable for a drug-delivery vector compared with therapeutic antibodies. SCF has been reported as an effective delivery vector towards KIT-positive neuroblastomas and colorectal cancer cells with expression of wild-type KIT in vitro [29] but has not been tested in mutant-KIT-driven malignancies. In the present study, we evaluated SCF as an effective vector to target mutant-KIT-driven GIST cancer cells, and the conjugated drug SCF-DM1 was tested for its efficacy in mutant-KIT-driven GIST cells in vitro and in vivo with a xenograft mouse model.

## Methods

### Cell lines and reagents

THP-1 (TIB-202) cell line was obtained from ATCC (Manassas, USA) and cultured in RPMI 1640 medium plus 10% fetal bovine serum (FBS). GIST cell lines (GIST T1 with KIT V560_Y578del, GIST 882 with KIT M541L/K642E, and GIST 430 with KIT V560_L576del/V654A) were cultured in IMDM medium plus 10% FBS. Mutations of *KIT* in these three cell lines were verified by Sanger sequencing. Primary KIT-positive GIST cells were from dissected tumor samples of GIST patients by gentle MACS Dissociator (Miltenyi Biotec, Bergisch Gladbach, Germany). Patients were provided with written informed consent using a protocol approved by the Institutional Review Board of the Seventh Affiliated Hospital, Sun Yat-sen University, in accordance with the Declaration of Helsinki. BL21 (DE3) *E. coli* and pET20 vector were products of Merck (Kenilworth, USA). DM1, sulfo-SMCC (sulfosuccinimidyl-4-(N-maleimidomethyl)cyclohexane-1-carboxylate) and imatinib mesylate were purchased from MCE (Monmouth, USA). Anti-His polyclonal antibody and rhSCF expressed in Sf9 cells were purchased from Sino biological (Beijing, China).

#### Expression and purification of rhSCF

A DNA fragment encoding the ectodomain of human SCF (NM_003994.6, amino acids 26–186) with a C-terminal 8 × His tag was amplified with primers For-SCF_ecto_ (5′-gcatccatggAAGGGATCTGCAGGAATCGT) and Rev-SCF_ecto_ (5′-ttatctcgagTTAGTGGTGGTGGTGATGATGATGGTGTAGGCTGGAGTCTCC) from a THP-1 cDNA library. The product was cloned in frame with a pelB signal peptide sequence into pET20 vector and then verified by Sanger sequencing. Recombinant pET20-SCF/BL21 (DE3) cells were cultured in ZYM-505 medium for 8 h at 37 °C. This bacterial culture was then inoculated and auto-induced in ZYM-5052 medium for 24 h at 28 °C [30]. Periplasmic fraction of collected bacteria pellet containing rhSCF was obtained by using the osmotic shock method [31] and was then mixed 1:1 (v/v) with buffer T (40 mM sodium phosphate buffer (PB), 0.6 M NaCl, 60 mM imidazole, pH 8.0). This diluted periplasmic sample was then loaded onto a Ni–NTA Sepharose column pre-equilibrated with buffer M (20 mM PB, 0.5 M NaCl, 35 mM imidazole, pH 8.0). After sample loading, the column was washed with 6 column volumes (CV) of buffer M and 8 CV of buffer P (20 mM PB, 0.5 M NaCl, 75 mM imidazole, pH 8.0) and then eluted with buffer Q (20 mM PB, 0.5 M NaCl, 800 mM imidazole, pH 6.5). Ammonium sulfate was added to the eluted fraction to a concentration of 0.78 M and gently stirred for 10 min. The sample solution was centrifuged at 12,000 rpm for 20 min, and the supernatant was loaded into a phenyl Sepharose column pre-equilibrated with buffer R (20 mM PB, pH 8.0, 0.78 M ammonium sulfate). The flow-through fraction containing rhSCF was collected and was exchanged to buffer S (0.04 M PB, 0.15 M NaCl, 1 mM EDTA, pH 7.10) by using Minimate EVO system (membrane cutoff value 5 kDa) (Pall, Washington, USA) through repeatedly dilution and ultrafiltration to remove NH_4_^+^ ion. This final samples from the purification steps were analyzed by polyacrylamide gel electrophoresis (SDS-PAGE) and immunoblotting. Bioactivity of purified rhSCF was tested in cell-based assays.

### Conjugation of rhSCF with DM1

Purified rhSCF and DM1 were conjugated via the chemical linker sulfo-SMCC. Sulfo-SMCC powder was dissolved in ultrapure water to form the stock solution (4.8 mg/mL) immediately before use. Protein concentration of rhSCF in buffer S was adjusted to 1 mg/mL. Molar ratio between sulfo-SMCC and rhSCF was 20:1. Sulfo-SMCC stock solution (10 mM in DMA) was added to the rhSCF solution. The mixture was gently agitated and incubated at room temperature for 1 h, and was then exchanged to buffer S by an ultrafiltration tube (cutoff value 5 kDa) to remove the unconjugated sulfo-SMCC. Before addition of DM1 stock solution (10 mM in DMA) into SCF-SMCC, a 10% (v/v) final concentration of DMA was added into SCF-SMCC solution to facilitate DM1 solvation. The DM1 stock solution was then added to the SCF-SMCC solution at a molar ratio of 20:1 between DM1 and SCF-SMCC. The mixture reacted for 2 h at room temperature. Then, the reaction product was centrifuged to remove precipitation, and the supernatant was exchanged to sodium phosphate buffer (0.15 M NaCl, 20 mM sodium phosphate buffer, pH 7.2, PBS) by rounds of centrifugation in an ultrafiltration tube (cutoff value 5 kDa) (Millipore, Burlington, USA) to remove unconjugated small molecule drugs and linkers. The theoretical concentration of unconjugated DM1 was controlled below 10 pM. The final product, SCF-SMCC-DM1 (abbreviated as SCF-DM1), was stored at − 20 °C. SCF-DM1 was analyzed by reducing SDS-PAGE to primarily characterize the conjugated product. SCF-DM1 conjugation product was also analyzed by LC–MS. Briefly, SCF-DM1 was separated on ACQUITY UPLC Protein BEH C4 Column by Ultimate 3000 UPLC (Thermo Fisher Scientific, Waltham, USA) and detected by AB SCIEX TripleTOF 5600 Mass Spectrometer (AB SCIEX, Framingham, USA). Bioactivity of SCF-DM1 was tested in cell-based assays.

### Bioactivity assay of rhSCF

For bioactivity assays, 1 × 10^6^ THP-1 cells were resuspended in culture medium without FBS and plated per well in a 6-well plate. After overnight incubation, 100 ng/mL rhSCF, 100 ng/mL rhSCF reference standard, or 100 ng/mL SCF-DM1 in plain culture medium was added. Cells were collected at different time points (10, 30, and 60 min) and lysed in cell lysis buffer. Samples were separated by SDS-PAGE and analyzed by immunoblotting. For internalization assay, 5 × 10^5^ cells were cultured in complete culture medium per well in a 12-well plate. Either 100 nM DM1, 100 ng rhSCF, or 100 ng/mL SCF-DM1 was added to the culture medium and incubated for 1 h. Cells were collected, washed twice with cold washing buffer W (1% BSA, 0.03% Proclin-300 in PBS), and then stained with APC-conjugated anti-KIT antibody on ice for 1 h. After three times washes with cold buffer W, cells were resuspended in buffer W and analyzed by a CytoFlex LX flow cytometer (Beckman Coulter, Brea, USA). Flow data was analyzed by FlowJo V10 software (FlowJo, Ashland, USA).

### Functional assay of SCF-DM1

For the cell viability assays, 3 × 10^4^ cells were plated per well in a 96-well plate with complete culture medium. For the treatment group, drugs were added to the corresponding wells with the final concentration from 0.05 nM to 100 nM for SCF-DM1 and 0.1 nM to 1 μM for DM1 and imatinib. After 5 days, cell viability was measured by the cell counting kit-8 method, and absorbance was measured with a BioTek synergy H1 microplate reader (BioTek, Winooski, USA).

For apoptosis analysis by immunoblotting, 1 × 10^6^ GIST cells were cultured with different treatment (SCF-DM1: 25 nM and 50 nM, DM1: 250 nM and 500 nM, imatinib: 500 nM) for 3 days. Protein samples were separated by SDS-PAGE and analyzed by immunoblotting. Antibodies recognizing cleaved CASPASE-3 and cleaved PARP were used as primary antibodies to detect apoptosis.

For apoptosis analysis by FACS, 1 × 10^6^ GIST cells were treated with different drugs (SCF-DM1: 50 nM or 100 nM, DM1: 500 nM, imatinib: 250 nM, 500 nM or 1 μM) for 3 days and then stained with APC-annexin V and propidium iodide (PI) before detection by FACS.

For cycle analysis of cells, 1 × 10^6^ GIST cells were treated with different drugs (SCF-DM1: 50 nM, DM1: 200 nM) for 48 h. Collected cells were washed with PBS and resuspended in 1 mL of 70% ethanol for at least 4 h at − 20 °C. After washed by PBS and resuspended in PI solution (0.1% Triton X-100, 0.1 mg/mL DNase-free RNase, 2 μg/mL PI, in PBS), samples were analyzed on a CytoFlex LX flow cytometer.

For colony assays, two hundred GIST T1 or GIST 430 cells were plated per well in a 6-well plate with 50 nM SCF-DM1, 500 nM, or 1000 nM imatinib or vehicle. Cells were cultured for 2 weeks, and colonies were fixed in 10% formalin solution for 10 min and stained with 0.01% (w/v) crystal violet solution for 30 min before counting with microscopy.

### Efficacy of SCF-DM1 in a cell line-derived xenograft mouse model

Female SCID beige mice (5 weeks) were purchased from Charles River Laboratories (Wilmington, USA). Animal care and experiments were performed in accordance with the ARRIVE guidelines. 2 × 10^6^ GIST 430 cells in PBS mixed with 50% of Matrigel were injected subcutaneously into the upper flank of mice. When tumor volumes were between 100 and 200 mm^3^, mice were randomly separated into SCF-DM1 group (*n* = 3), DM1 group (*n* = 3), imatinib group (*n* = 3), and control group (*n* = 3). SCF-DM1 (50 μg) or DM1 (8.35 μg, 5 times of DM1 in 50 μg of SCF-DM1) was intratumorally administrated. Imatinib (100 mg/kg/day) was orally administrated. Control was administrated with PBS intratumorally. Body weight and tumor size were measured every three days. Tumor volume was calculated as (length × width^2^)/2. Mice with tumor volumes exceeding 1000 mm^3^ were sacrificed and excised tumors were stored in 10% formaldehyde solution.

### Statistics

Data were displayed as the mean ± standard deviation. Data in each group passed the normality test. Difference between two groups was calculated using unpaired *t*-test. Differences among multiple groups were calculated using one-way ANOVA. Significance was analyzed by Origin Pro 9.0 software (Originlab, Northampton, USA), and *p* < 0.05 was considered significant.

## Results

### Bioactive rhSCF was expressed and purified from recombinant *E. coli* cells

**Fig. 1:**
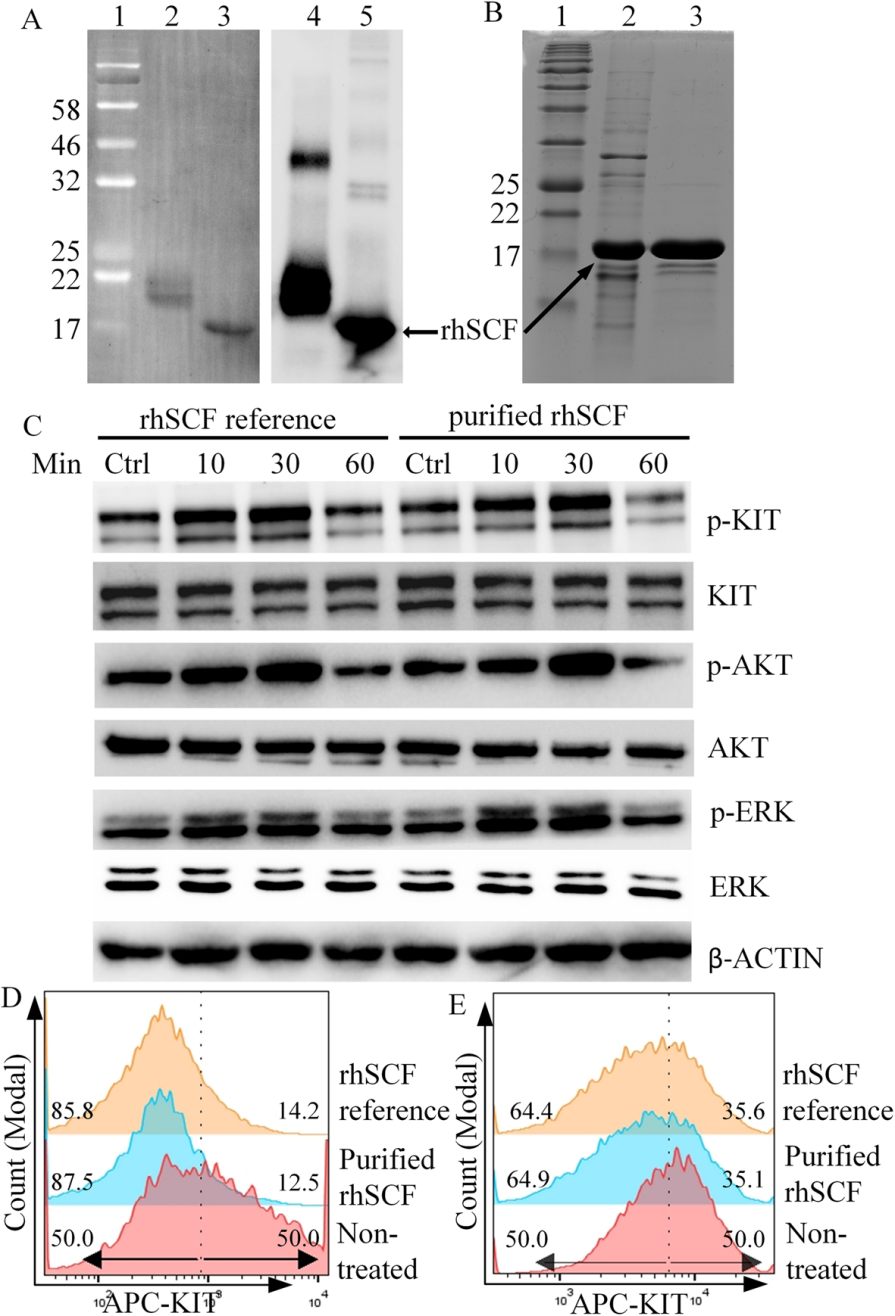
Expression, purification, and bioactivity evaluation of rhSCF. **A** Immunoblotting analysis of purified rhSCF protein. Lane 1: pre-stained protein marker; lanes 2 and 4: reference rhSCF from Sf9 cells. Due to glycosylation modification, rhSCF from Sf9 cells has a higher molecule weight than rhSCF without glycosylation; lanes 3 and 5: purified rhSCF. Left panel: PVDF membrane captured by using stain free method on Bio-Rad ChemiDoc system. Right panel: western blot result of rhSCF reference and purified rhSCF. **B** SDS-PAGE result of rhSCF after purification. Lane 1: protein marker; lane 2: sample after Ni–NTA Sepharose purification; lane 3: sample after phenyl Sepharose purification. **C** Immunoblot analysis of phosphorylation status of KIT signaling proteins in THP-1 treated by rhSCF (100 ng/mL). **D**, **E** Membrane KIT expression levels in THP-1 (**D**) and GIST 430 (**E**) treated by rhSCF (100 ng/mL). The vertical dot lines in **D** and **E** were given to view the induction effect of SCF. Numbers indicated left-side or right-side cell population (%) separated by dot lines in each group

SCF ectodomain gene was successfully obtained from the cDNA library of THP-1, and verified by Sanger sequencing. Recombinant human SCF protein was expressed and purified from periplasm of induced recombinant BL21 (DE3) *E. coli*/pET20-SCF. Purified rhSCF was detected by anti-His tag antibody (Fig. 1A), indicating the soluble form of rhSCF expressed in *E. coli*. rhSCF was purified by Ni–NTA Sepharose and phenyl hydrophobic Sepharose to about 95% purity (Fig. 1B). About 0.18 mg of purified rhSCF was obtained from 1 L of fermentation broth. We further detected the bioactivity of rhSCF in bioactivity assay and internalization assay. Binding of SCF to KIT induces the phosphorylation of KIT, ERK, and AKT. In THP-1 with wild-type KIT, our rhSCF induced phosphorylation of KIT, ERK, and AKT to similar levels as the commercial rhSCF from Sf9 (Fig. 1C), indicating the bioactivity of our rhSCF to activate KIT signaling pathway. Also, our rhSCF induced the internalization of KIT to similar levels as commercial rhSCF from Sf9 in THP-1 and GIST 430 (Fig. 1D, E). These results demonstrated that rhSCF purified from *E. coli* was functional, which was used for following studies.

### Conjugation of rhSCF and DM1

**Fig. 2:**
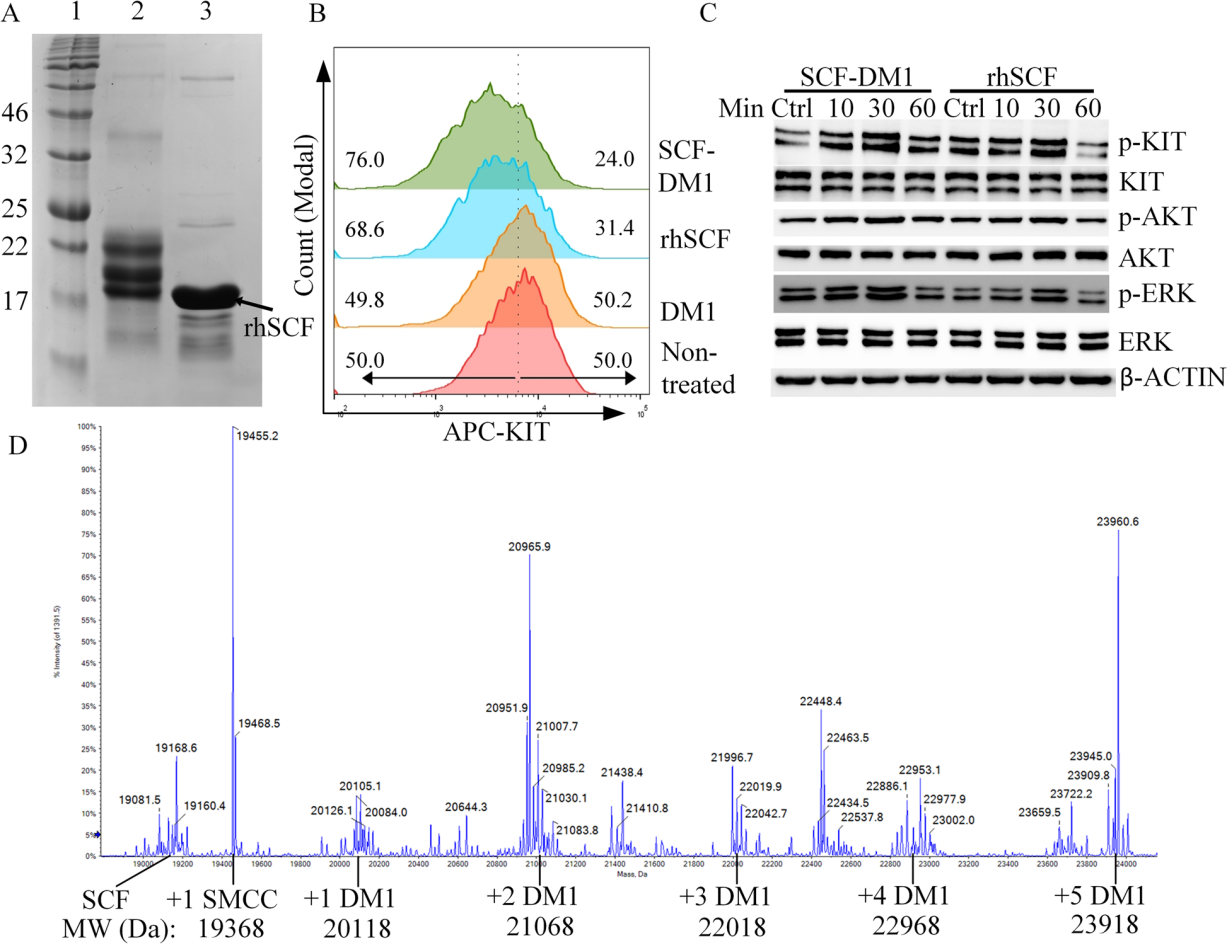
Preparation and bioactivity evaluation of SCF-DM1 A. SDS-PAGE result of SCF and DM1 conjugation product. Lane 1: protein marker; lane 2: SCF and DM1 conjugation product; lane 3: rhSCF. **B** Membrane KIT expression levels in GIST T1 cells with treatment of drugs. Vertical dot line in **B** was given to view the induction effect of drugs, and numbers indicated left-side or right-side cell population (%) separated by the dot line in each group. SCF-DM1: 100 ng/mL, rhSCF: 100 ng/mL, DM1: 100 nM. **C** Immunoblot analysis of phosphorylation status of KIT signaling proteins in THP-1 cells with treatment of drugs. rhSCF: 100 ng/mL; SCF-DM1: 100 ng/mL. **D** LC–MS result of SCF and DM1 conjugation product. + X DM1 means one molecule of SCF was conjugated with X molecule(s) of DM1, + 1 SMCC means one molecule of SCF was conjugated with 1 molecule of SMCC

rhSCF was conjugated with DM1 via the SMCC chemical linker. When SCF-SMCC reacted with DM1, 10% DMA was added to prevent the precipitation of DM1 in water system; meanwhile, the denaturation of rhSCF caused by DMA was minimized. The final reaction product SCF-DM1 was a mixture and had higher molecule weights than rhSCF (Fig. 2A). Here, we assumed that one molecule of DM1 was conjugated with one molecule of SCF for the calculation of molar concentration of SCF-DM1 based on our previous study [22]. After conjugation, SCF-DM1 was exchanged to PBS, and unconjugated DM1 was removed by rounds of ultrafiltration. To check if this conjugation product was functional, the above bioactivity assay and internalization assay were performed. The internalization assay showed that SCF-DM1 was capable of inducing internalization of membrane KIT, while DM1 alone had no such effect (Fig. 2B). Bioactivity assay was performed on THP-1 cells with overnight serum starvation. The phosphorylation level of KIT elevated with the stimulation of SCF-DM1 and rhSCF, indicating that SCF-DM1 maintained the bioactivity of rhSCF (Fig. 2C). These results demonstrated that rhSCF maintained its bioactivity after conjugation with DM1. LC–MS confirmed that reaction product was nonhomogeneous, with 1–5 molecule(s) of DM1 conjugated to one molecule of rhSCF (Fig. 2D).

### SCF-DM1 inhibited the proliferation of GIST cells

**Fig. 3:**
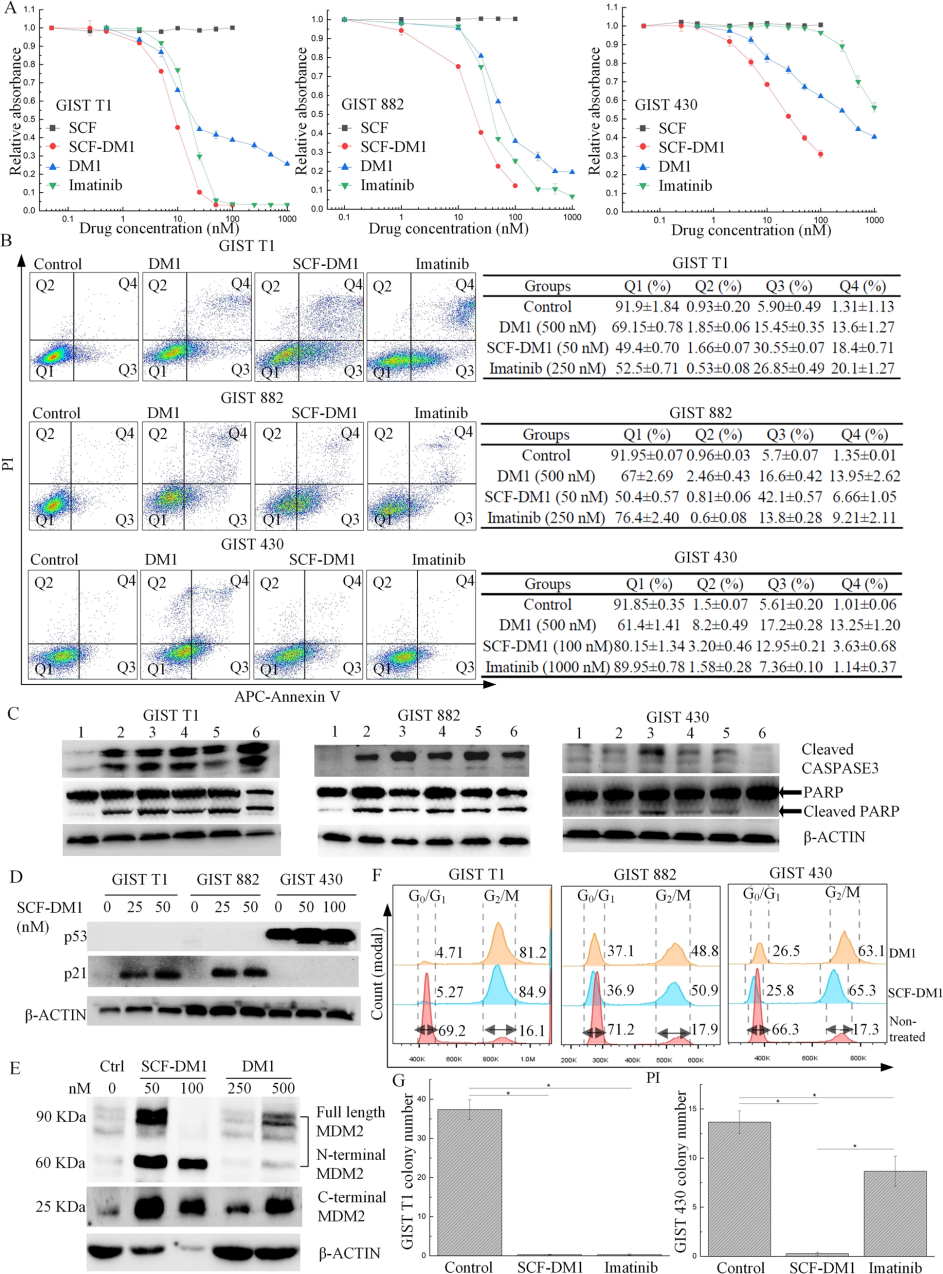
Cytotoxicity effect of SCF-DM1 on GIST cell lines. **A** Cell proliferation assay of GIST cells with treatment of drugs. **B** FACS detection of apoptosis in GIST cells with treatment of drugs. **C** Immunoblot analysis of apoptosis-related proteins of GIST cells with treatment of drugs. Lane 1: control; lane 2: SCF-DM1 25 nM; lane 3: SCF-DM1 50 nM; lane 4: DM1 250 nM; lane 5: DM1 500 nM; lane 6: imatinib 500 nM. **D** Immunoblot analysis of p53 and p21 protein in GIST cells with treatment of SCF-DM1. **E** Total MDM2 expression of GIST 430 cells with treatment of drugs for 72 h. **F** Cell cycle analysis of GIST cells with treatment of drugs. Numbers indicated G_0_/G_1_ or G_2_/M population (%). SCF-DM1: 50 nM, DM1: 200 nM. **G** Clonogenic assay of GIST cells with treatment of drugs. SCF-DM1: 50 nM; imatinib: 500 nM for GIST T1, 1000 nM for GIST 430

SCF-DM1 was tested for its efficacy on GIST cells. In cell viability assay, SCF-DM1 showed inhibitory activity in GST T1, 882, and 430 cells (Table 1, Fig. 3A). Note that SCF-DM1 had higher potency in these cells than unconjugated DM1 alone. Imatinib effectively inhibited the growth of imatinib-sensitive GIST cells but required much higher concentration to inhibit GIST 430, the imatinib-resistant cells. The apoptosis assay also showed that SCF-DM1 increased annexin V-positive population in three GIST cell lines (Fig. 3B).

**Table 1:** IC_50_ of three drugs on GIST cell lines

Drug (nM)	SCF-DM1	DM1	Imatinib
GIST T1	9.04	10.41	16.54
GIST 882	18.21	46.56	38.24
GIST 430	29.96	341	1155

All three drugs induced elevated levels of apoptosis-related proteins, cleaved PARP, and cleaved CASPASE3 in imatinib-sensitive GIST T1 and 882 cells. In imatinib-resistant GIST 430 cells, SCF-DM1 and DM1 alone induced elevated cleaved PARP, but cleaved CASPASE3 was limitedly induced compared with imatinib-sensitive GIST cells. In contrast, imatinib failed to induce cleaved PARP and cleaved CASPASE3 as expected (Fig. 3C). We also analyzed p53 expression level in three GIST cell lines with or without treatment of SCF-DM1. In imatinib-sensitive GIST cells, p53 cannot be detected with treatment of these drugs (Fig. 3D). However, the expression level of p53 was high in GIST 430 cells with or without treatment of SCF-DM1 (Fig. 3D). p21 is the downstream effector of p53 protein upon DNA damage, which increased in imatinib-sensitive GIST cells treated with SCF-DM1 but was undetectable in GIST 430 cells (Fig. 3D). Sequencing of *TP53* gene in GIST 430 showed the full length p53 with P72R polymorphism [32]. Downstream effector MDM2 of p53 in GIST 430 cells significantly increased with treatment of drugs (Fig. 3E). These results indicated that downstream signaling pathway of p53 was functional in GIST 430 cells, as the expression of total MDM2 greatly increased in response to the treatment of SCF-DM1 and DM1, but the ability of p53 to induce apoptosis was impaired in GIST 430 cells when treated by SCF-DM1 and DM1.

**Fig. 4:**
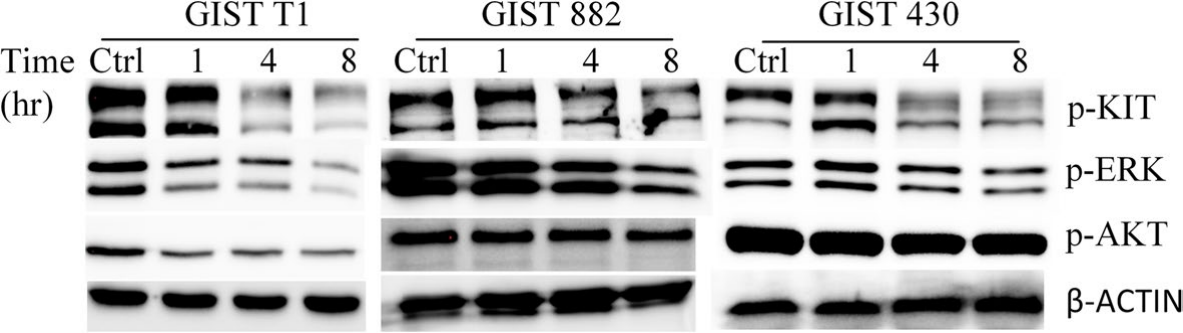
Immunoblot analysis of phosphorylation status of KIT signaling proteins in GIST cell lines with treatment of SCF-DM1 (100 ng/mL). Cells were cultured in IMDM medium plus 10% FBS

Cell cycle analysis showed that SCF-DM1 induced cell cycle arrest at G2/M phase (Fig. 3E). Colony assay was also utilized to measure drug efficacy. For GIST T1 cells, 50 nM of SCF-DM1 or 500 nM of imatinib fully inhibited the colony forming ability. For GIST 430 cells, 1 μM of imatinib only reduced the colony numbers by about 40% compared with control group. In contrast, 50 nM of SCF-DM1 fully inhibited the forming of colonies (Fig. 3F). KIT mutations in GIST cell lines do not interfere with the ligand-receptor binding interface, which retain the ability to bind with SCF. Compared with control, phosphorylation level of KIT, ERK, and AKT decreased after hours incubation in the complete culture medium with SCF-DM1 in all three GIST cell lines (Fig. 4). This result demonstrated that SCF-DM1 did not activate but inhibited KIT signaling in these GIST cells.

### SCF-DM1 specifically targeted KIT-expressing HCD57 cells

**Fig. 5:**
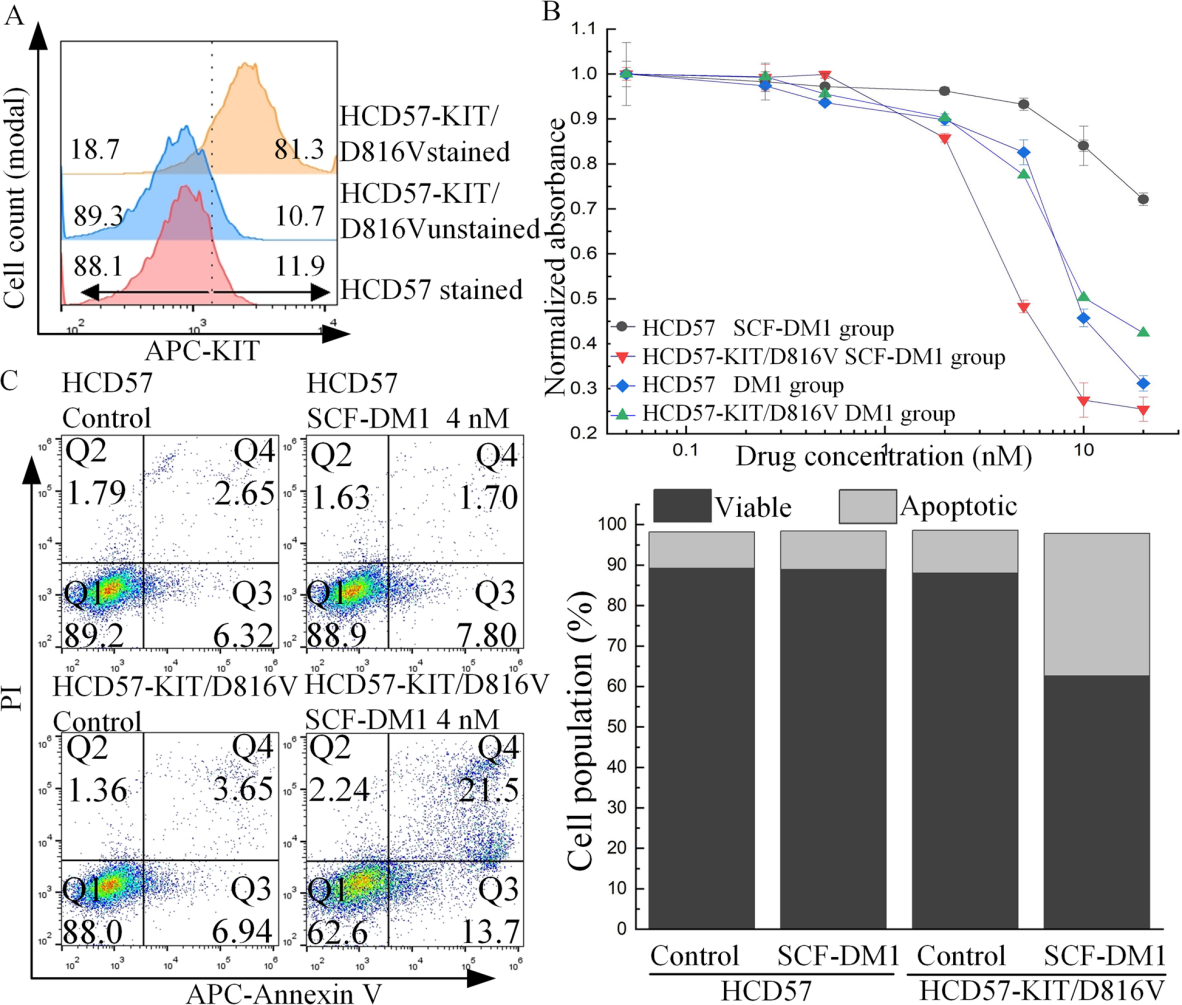
Specificity of SCF-DM1. **A** Membrane KIT expression level of stable HCD57-KIT/D816V cells. Vertical dot line in **A** was given to view the induction effect of drugs. Numbers indicated left-side or right-side cell population (%) separated by the dot line in each group. **B** Cell inhibition assay of HCD57 cells with treatment of drugs for 72 h. C. FACS analysis and bar chart analysis of apoptosis in GIST cells with treatment of drugs for 72 h

HCD57 cells, a murine erythropoietin (EPO)-dependent erythroleukemia cell line [33], were utilized to test the specificity of SCF-DM1. HCD57 cells were transfected with pMSCV-GFP-KIT/D816V plasmid, and cells were selected in culture medium without EPO. After 2 weeks, surviving cells were subcloned in MethoCult™ medium (Stemcell Technologies, Vancouver, Canada). One clone of HCD57-KIT/D816V was used for the specificity assay of SCF-DM1 (Fig. 5A). In HCD57-KIT/D816V cells, IC_50_ of SCF-DM1 was 3.6 nM, while in parental HCD57 cells, IC_50_ of SCF-DM1 was > 100 nM (Fig. 5B). FACS demonstrated that HCD57-KIT/D816V had 24.61% increment of annexin V + population of cells treated with SCF-DM1, compared with negligible increment in parental HCD57 treated by SCF-DM1 (Fig. 5C). The above results indicated the specificity of SCF-DM1 towards KIT-positive cells.

### SCF-DM1 inhibited primary GIST cells ex vivo

**Fig. 6:**
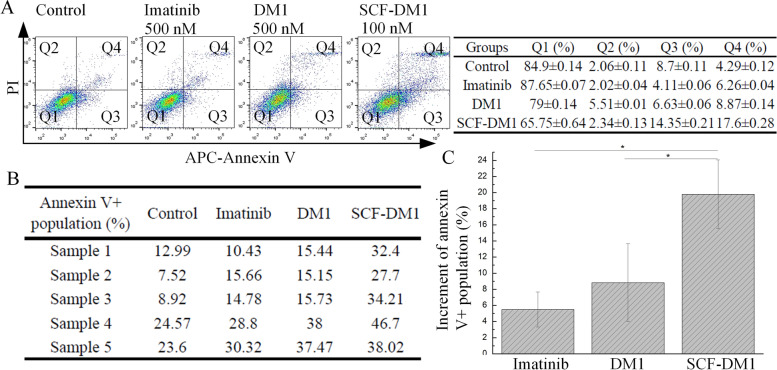
Effect of SCF-DM1 on primary GIST cells. **A** FACS analysis of primary GIST cells with treatment of drugs. **B** Annexin V positive population of five primary GIST cells with treatment of drugs by FACS analysis. Sample 1: GIST located at stomach; samples 2–5: GIST tumor metastasized in liver. **C** Bar chart of increment of Annexin V + population in primary GIST patient cells with treatment of different drugs after deduction of control. Data was displayed in mean ± standard deviation

The efficacy of SCF-DM1 was also tested with primary GIST cells. Samples were all confirmed to be KIT positive. All GIST samples were imatinib-resistant. One was from GIST tumor in stomach; the others were from GIST tumors metastasized in liver. Samples were extracted by experienced gastrointestinal surgeons on excised tumor tissue. Single cells were obtained from tumor samples by human tumor dissociation kit on a gentle MACS dissociator. Cells were treated with SCF-DM, DM1, and imatinib for 4 days before measured by FACS. A FACS result of primary GIST cells with different treatment of drugs was shown (Fig. 6A). SCF-DM1 generally had better apoptosis-inducing ability compared with DM1 alone or imatinib on primary GIST cells (Fig. 6B, C). The results demonstrated SCF-DM1 was effective to induce apoptosis in primary GIST cells ex vivo and was effective in imatinib-resistant metastasized GIST cells.

### Efficacy of SCF-DM1 in xenograft mouse model

**Fig. 7:**
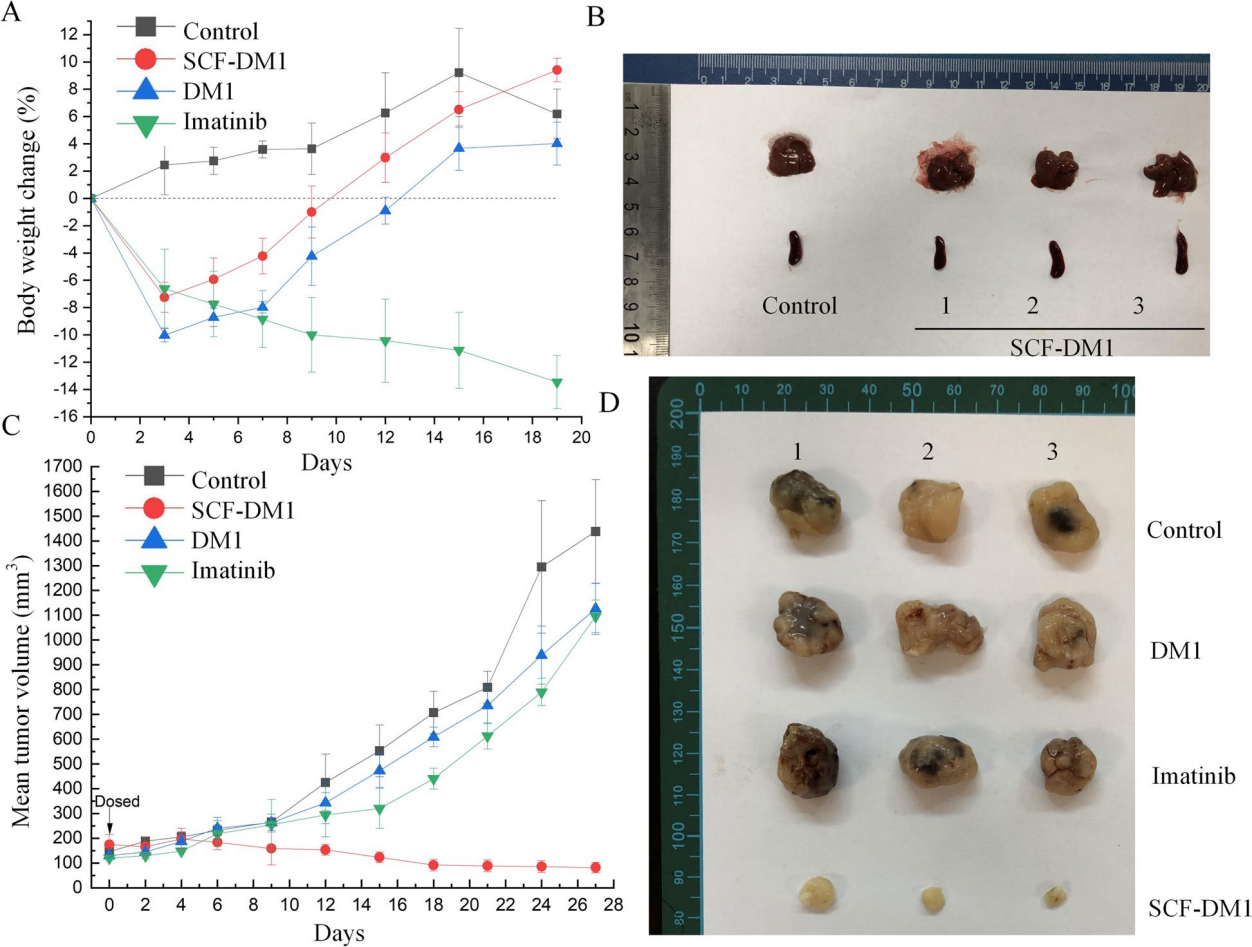
Efficacy of SCF-DM1 on the mouse model. **A** Body weight change of mice with drug administration. **B** Liver and spleen of mice treated with SCF-DM1. **C** Tumor volume of mice with treatment of different drugs. Single injection of SCF-DM1 (50 μL, 1 mg/mL) and DM1 was intratumorally administrated on day zero. Imatinib (100 mg/kg) was given once per day until the mice were sacrificed. **D** Tumors dissected from mice

The SCF-DM1 efficacy was evaluated in vivo in SCID beige mice engrafted with GIST 430 cells. We delivered SCF-DM1 intratumorally. Upon SCF-DM1 injection, the body weight decreased by about 7.2% in first 3 days, then recovered and started to gain weight in following days (Fig. 7A). The volume and appearance of liver and spleen in SCF-DM1 drug group were similar to that of control group (Fig. 7B). These results indicated that SCF-DM1 was well tolerated in this mouse model with current dosing scheme. Compared with other groups, SCF-DM1 significantly inhibited the growth of GIST 430-derived tumors in a 27-day observation (Fig. 7C, D). In contrast, imatinib and DM1 had limited effect of growth inhibition on GIST 430-derived tumors (Fig. 7C, D). These results showed that SCF-DM1 was capable of inhibition of imatinib-resistant GIST cells in vivo in the GIST xenograft mouse model.

Also, we performed a preliminary experiment to evaluate SCF-Fc fusion protein as the vector to deliver DM1 into GIST cells in vivo via intravenous administration. We conjugated SCF-Fc with DM1 (Figure S1. A) and injected the conjugation products SCF-Fc-DM1 intravenously into GIST mouse model at 6.0 mg/kg once. We observed that the proliferation of GIST 430 cells was inhibited significantly in mice injected with SCF-Fc-DM1 on day 27, compared with controls (Figure S1. C and D). This preliminary study demonstrated that SCF can be used as an effective vector that can be improved by protein engineering to target KIT-mutant GIST.

## Discussion

**Fig. 8:**
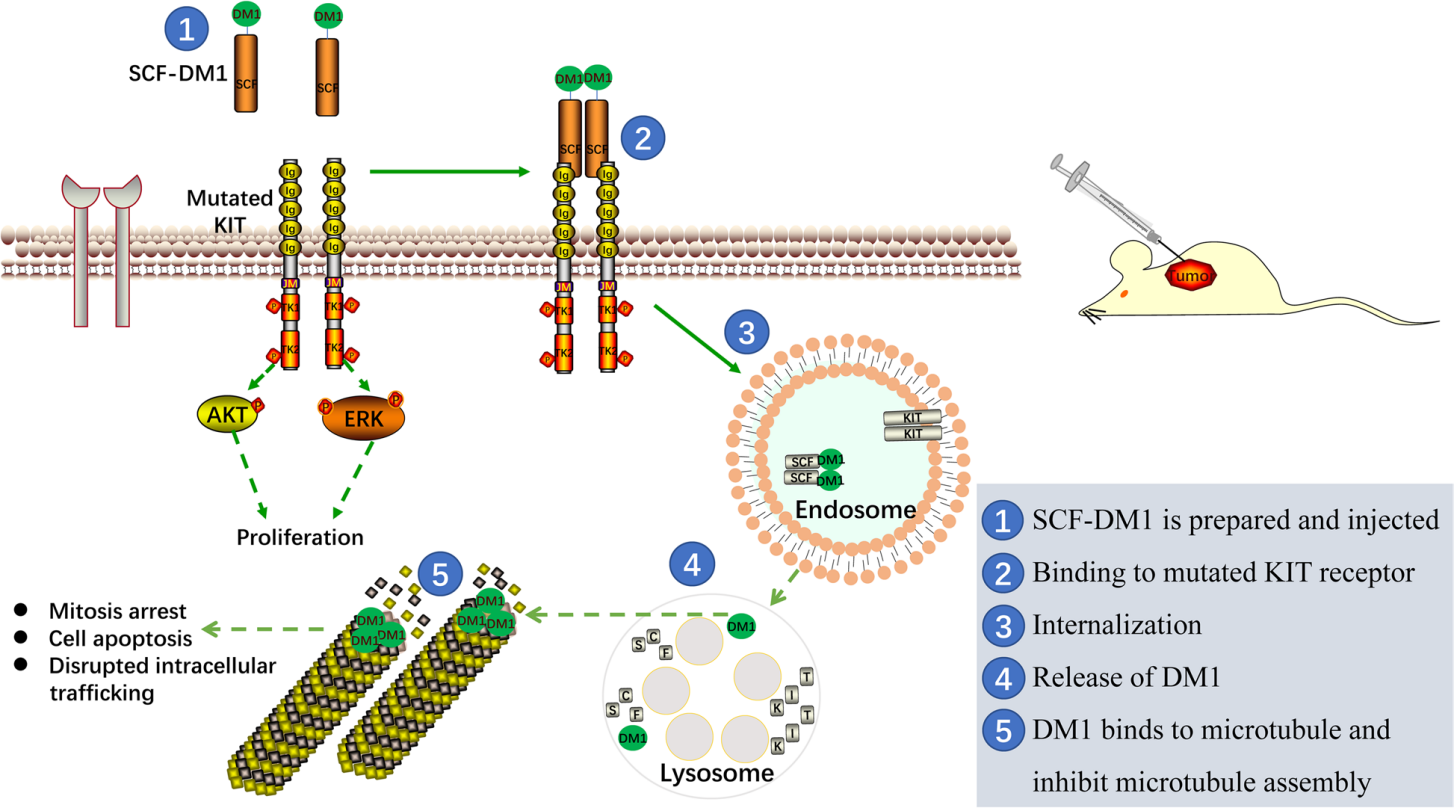
Schematic diagram of the effects of SCF-DM1 on KIT-mutated GIST cells

Majority of GIST malignant cells depend on the KIT signaling pathway for survival. Targeting KIT therapy has achieved success, as imatinib significantly prolongs the 5-year survival of GIST patients [34]. However, GIST cells that contain other forms of activating-mutations, gradually accumulate during the imatinib adjuvant therapy process and render resistance to imatinib therapy [35]. Here, we presented SCF-based drug conjugate, SCF-DM1 showed inhibitory activity (IC_50_ < 30 nM) towards KIT-mutated GIST cells in vitro and showed inhibitory effect against imatinib-resistant GIST 430 cells in a mouse model. Compared with imatinib, SCF-DM1 showed efficacy towards both imatinib-sensitive and -resistant GIST cells in vitro. In primary imatinib-resistant GIST tumor cells, SCF-DM1 induced significant apoptosis than DM1 and imatinib. These results showed that SCF was an effective vector to deliver conjugated drugs into cell cytosol, and SCF-DM1 was a possible drug against KIT-positive GIST by targeting mutated-KIT (Fig. 8).

The resistance of GIST cells against imatinib has been widely reported to be the result of secondary mutations of KIT, which cause activation of KIT in the presence of imatinib [34, 36]. GIST 430 is imatinib-resistant GIST cell line. With treatment of DM1 or SCF-DM1, the majority of GIST 430 cells were still alive, but their cell cycle was arrested. The proliferation was well-inhibited, but no evident apoptosis was observed. Protein expression level of p53 in GIST 430 cells is high, and the p53-mediated signaling pathway is functional in GIST 430 cells [32]. It was interesting to find that cleaved forms of MDM2 were both significantly increased in GIST 430 cells treated by SCF-DM1 or DM1. Cleavage of MDM2 has been reported to be caused by CASPASE-2 in the PIDD complex, which is also an authentic downstream effector of p53 [37]. The 60 kDa N-terminal MDM2 lacks the ubiquitin ligase function [38]. When N-terminal MDM2 binds with p53, it can protect p53 from degradation mediated by full length MDM2 [37]. This mechanism can lead to evasion of cancer cells from apoptosis mediated by p53 signaling. Further studies are needed to find whether p53-PIDD protects GIST 430 cells from apoptosis under treatment of SCF-DM1 or DM1.

In this study, we found that DM1 inhibited GIST cells with IC_50_ at nanomolar concentrations, which is different from several other reports [16, 17]. There are several reasons that can lead to the difference. Firstly, DM1 and its derived metabolites have different toxicities on cancer cells. S-Me-DM1, the most toxic metabolite of DM1, has a stronger inhibitory effect against cancer cells than free DM1 [39]. Therefore, S-Me-DM1 shows a lower IC_50_ against GIST cells [16] and KB cells [39], compared with free DM1 used in the present study. Secondly, the use of different methods to detect cell viability can contribute to variations of IC_50_ of DM1 on GIST cells. The luminescent method reported in other studies is more sensitive than CCK-8 method used in the present study [40]. These factors together could lead to variations in IC_50_ of DM1 on GIST cells reported here and in other studies [16, 17].

Current approved therapeutic antibodies against oncogenic receptor tyrosine kinases are antagonists, which inhibit the activation of receptors by blocking the binding of ligands to receptors [41, 42]. This inhibitory mechanism only applies to wild-type receptors, but not mutant ones, since the activation of mutant receptors is ligand-independent. For receptors with gain-of-function mutations, inhibitory antibodies can pose the therapeutic effect by induction of internalization of receptors, instead of ligand blocking [43]. For example, cetuximab is effective to inhibit NSCLC cell lines with mutant EGFR. The effect is mediated through the induction of EGFR internalization by cetuximab, followed by degradation of mutant EGFR in lysosome [43]. Therefore, the blockage of binding of ligands and the induction of receptor internalization are two important mechanisms of therapeutic effects of antibodies. The latter is more practical in cancer cells with mutant receptors, including GIST with gain-of-function KIT mutations.

Around 80% of GIST has gain-of-function KIT mutations. Therapeutic antibodies targeting mutated KIT function mainly by inducing internalization of receptor and Fc-mediated antibody function. There is one report related to therapeutic anti-mutated-KIT antibody against GIST [16]. LMJ729, a monoclonal antibody against KIT induces internalization of mutated KIT of GIST cells in vitro, but has little inhibition effect in GIST 430-derived xenograft mouse model [16]. This shows that LMJ729 does not have enough inhibition capability against GIST with KIT mutation. This phenomenon also happens in the anti-FLT3 therapeutic antibody IMC-EB10, which shows good efficacy against leukemia cells with mutated FLT3 in preclinical study [44], but fails to show efficacy in clinical phase 1 trial [45]. Therapeutic antibodies can meet with other problems which can restrict its clinical efficacy. First, therapeutic antibodies rely on the innate immune system to eliminate cancer cells. However, when antibodies and its targets form complex, they could be internalized by cells. This phenomenon decreases the membrane levels of antibodies; thus, the anchor sites for effector cells are reduced [46, 47]. Second, though antibody-target complex can successfully recruit immune cells, with unknown mechanism, immune cells can eliminate the complex by trogocytosis, not imposing the ADCC effect on the antibody-coated target cells [48]. Third, tumor environment has immune-suppressive cytokines or factors, which make immune effector cells anergic [49, 50]. GIST environment also has large quantity of TGF-β1, which can reduce the activity of immune effector cells [51].

One possible and practical strategy against malignancies caused by mutant oncogenic receptors (e.g., KIT, FLT3) is receptor-targeted proteins conjugated with cytotoxic drugs [20]. The most important aspect of this strategy is that receptor-targeted proteins must induce internalization of the mutant receptors. Antibody conjugated drug (ADC) targeting mutated KIT has been reported to be effective in GIST in preclinical studies [16, 17]. In this study, we showed that SCF-DM1 was effective against KIT-mutant GIST cells. And SCF-DM1 had an inhibitory effect on KIT signaling in KIT-mutant GIST cells instead of a stimulatory role (Fig. 4). In this situation, the ligand SCF worked with the same mechanism as antibodies in conjugated drugs. They can both specifically bind with KIT, induce internalization of the complex, inhibit KIT signaling, and deliver drugs into cytosol of cancer cells.

SCF, compared with anti-KIT Fab moiety, is natural, easy-to-get and non-immunogenic. The affinity between SCF and KIT is also good (10^−10^–10^−9^ M) [27, 28]. The bioproduction technology and cell lines (CHO, HEK293) used for production of antibodies are also suitable for the production of SCF. The favorable pharmacokinetics of antibodies are rendered by the Fc fragment. SCF can be easily fused with IgG Fc to have a comparable pharmacokinetics compared with antibodies [52, 53]. The efficacy of the Fc fusion form of SCF conjugated with DM1 was explored briefly in this study (Figure S1). With Fc tag, SCF-Fc-DM1 was capable to inhibit GIST cells engrafted in mice with a single injection intravenously, which may serve as an improved version of SCF drug conjugate in targeted therapy of GIST in the future. Also, the gene encoding SCF can be further mutated to obtain a better candidate for modified SCF-DM1 [27]. The principle of choosing the candidates could be based on affinity (K_D_ value), and/or receptor internalization rate. Finally, good candidates can be tested in the cell line-derived xenograft mouse model to choose the optimal one.

## Conclusions

Our proof-of-concept study showed that SCF was an effective vector for the delivery of cytotoxic drugs. SCF-DM1 demonstrated favorable efficacy towards GIST cell lines in vitro and primary GIST cells ex vivo. In GIST xenograft model, the results showed that SCF-DM1 was effective in inhibition the proliferation of GIST cells with favorable safety profiles in mice.

## Supplementary Information

**Additional file 1: Supplemental Figure S1.** The effect of SCF-Fc-DM1 on GIST 430 cells in mice. A. Conjugation of SCF-Fc and DM1. Lane1: pre-stained protein marker; Lane2: rhSCF-Fc protein; Lane3: rhSCF-Fc conjugated with SMCC (SCF-Fc-SMCC); Lane4: SCF-Fc-SMCC conjugated with DM1 (SCF-Fc-DM1). B. Effect of SCF-Fc-DM1 on GIST 430 cells *in vitro *by FACS analysis. C. Tumor volumes of mice treated with SCF-Fc-DM1 or FL-Fc-DM1 as the control. SCF-Fc-DM1 (6.0 mg/kg) and control (FL-Fc-DM1, 6.0 mg/kg) were administrated once on day zero. D. Tumors dissected from mice.

## Data Availability

All data generated or analyzed during this study are included in this published article and its supplementary information files.

## Abbreviations

rhSCF: Recombinant human stem cell factor
GIST: Gastrointestinal stromal tumors
GI: Gastrointestinal tract
DM1: Emtansine
JM: Juxtamembrane domain
TK: Tyrosine kinase
A-loop: Activation loop
ADC: Antibody drug conjugate
CV: Column volume
Sulfo-SMCC: Sulfosuccinimidyl-4-(N-maleimidomethyl)cyclohexane-1-carboxylate
DMA: Dimethylamine
HCD57-KIT/D816V: HCD57 cells stable transformed with *KIT/D816V* gene and expressed KIT/D816V protein
EPO: Erythropoietin
IC_50_: Half maximal inhibitory concentration
