# Supplementary material for: Efficacy of SCF drug conjugate targeting c-KIT in gastrointestinal stromal tumor
Source: BMC Med. 2022 Aug 24;20:257. doi: 10.1186/s12916-022-02465-3 (PMC9400206; doi:10.1186/s12916-022-02465-3)
Supplement: Supplementary file 1 — Additional file 1: Supplemental Figure S1. The effect of SCF-Fc-DM1 on GIST 430 cells in mice. A. Conjugation of SCF-Fc and DM1. Lane1: pre-stained protein marker; Lane2: rhSCF-Fc protein; Lane3: rhSCF-Fc conjugated with SMCC (SCF-Fc-SMCC); Lane4: SCF-Fc-SMCC conjugated with DM1 (SCF-Fc-DM1). B. Effect of SCF-Fc-DM1 on GIST 430 cells in vitro by FACS analysis. C. Tumor volumes of mice treated with SCF-Fc-DM1 or FL-Fc-DM1 as the control. SCF-Fc-DM1 (6.0 mg/kg) and control (FL-Fc-DM1, 6.0 mg/kg) were administrated once on day zero. D. Tumors dissected from mice. [file 12916_2022_2465_MOESM1_ESM.docx]

**SUPPLEMENTAL INFORMATION**

**Efficacy of SCF drug conjugate targeting c-KIT in gastrointestinal stromal tumor**

Dengyang Zhang^1*^, Chunxiao He^1*^, Yao Guo^1*^, Jianfeng Li^2^, Bo Li^1^, Yuming Zhao^1^, Liuting Yu^1^, Zhiguang Chang^1^, Hanzhong Pei^1^, Ming Yang^1^, Na Li^1^, Qi Zhang^1^, Yulong He^2^, Yihang Pan^1^, Zhizhuang Joe Zhao^3#^, Changhua Zhang^2#^, Yun Chen^1#^

1. Edmond H. Fischer Translational Medical Research Laboratory, Scientific Research Center, The Seventh Affiliated Hospital, Sun Yat-sen University, Shenzhen, 518107 Guangdong, China.

2. Center for Digestive Disease, The Seventh Affiliated Hospital, Sun Yat-sen University, Shenzhen, 518107, Guangdong, China.

3. Department of Pathology, University of Oklahoma Health Sciences Center, Oklahoma City, OK 73104, USA.

#Correspondence: Dr. Yun Chen, Edmond H. Fischer Translational Medical Research Laboratory, Scientific Research Center, The Seventh Affiliated Hospital, Sun Yat-sen University, Shenzhen, 518107 Guangdong, China; phone: (0755)81207021; email: cheny653@mail.sysu.edu.cn

Dr. Changhua Zhang, Center of Digestive Diseases, The Seventh Affiliated Hospital of Sun Yat-sen University, Shenzhen, Guangdong 518107, China, email: zhchangh@mail.sysu.edu.cn

Dr. Zhizhuang Joe Zhao, Department of Pathology, University of Oklahoma Health Sciences Center, Oklahoma City, 73104, USA, email: joe-zhao@ouhsc.edu

^*^These authors contributed equally to this work.

**Supplemental** **Methods**

**Conjugation of rhSCF-Fc with DM1**

rhSCF-Fc and DM1 were conjugated via the chemical linker sulfo-SMCC. Sulfo-SMCC powder was dissolved in ultrapure water to form the stock solution (4.8 mg/mL). Protein concentration of rhSCF-Fc in buffer S (0.04 M PB, 0.15 M NaCl, 1 mM EDTA, pH 7.10) was adjusted to 1 mg/mL. Molar ratio between sulfo-SMCC and rhSCF-Fc was 20:1. The mixture was gently agitated and incubated at room temperature for 1 hour, and was then exchanged to buffer S by an ultrafiltration tube (cutoff value 5 kDa) to remove the unconjugated sulfo-SMCC. The DM1 stock solution (10 mM in DMA) was then added to the SCF-Fc-SMCC solution at a molar ratio of 20:1 between DM1 and SCF-Fc-SMCC. The mixture reacted for 2 hours at room temperature. The supernatant was exchanged to sodium phosphate buffer (0.15 M NaCl, 20 mM sodium phosphate buffer, pH 7.2, PBS) by rounds of centrifugation in an ultrafiltration tube (cutoff value 5 kDa) (Millipore, Burlington, USA) to remove unconjugated small molecule drugs and linkers. The theoretical concentration of unconjugated DM1 was controlled below 10 pM. The final product, SCF-Fc-SMCC-DM1 (abbreviated as SCF-Fc-DM1), was stored at -20 °C. SCF-Fc-DM1 was analyzed by reducing SDS-PAGE to primarily characterize the conjugated product.

**Apoptosis analysis of GIST 430 cells under treatment of SCF-Fc-DM1**

1 × 10^6^ GIST 430 cells were treated with SCF-Fc-DM1 for 3 days and then stained with APC-annexin V and propidium iodide (PI) before detection by FACS.

**Efficacy of SCF-Fc-DM1 in a cell line-derived xenograft mouse model**

Female SCID beige mice (5 weeks) were purchased from Charles River Laboratories (Wilmington, USA). Animal care and experiments were performed in accordance with the ARRIVE guidelines. 2 × 10^6^ GIST 430 cells in PBS mixed with 50% of Matrigel were injected subcutaneously into the upper flank of mice. When tumor volumes were between 100-200 mm^3^, mice were randomly separated into SCF-Fc-DM1 group (n=3) and FL-Fc-DM1 control group (n=3). SCF-Fc-DM1 (6.0 mg/kg) or FL-Fc-DM1 (6.0 mg/kg) was intravenously administrated once at day zero. Body weight and tumor size were measured every three days. Tumor volume was calculated as (length × width^2^)/2. Mice with tumor volumes exceeding 1000 mm^3^ were sacrificed and excised tumors were stored in 10% formaldehyde solution.

**Supplemental** **Figures**


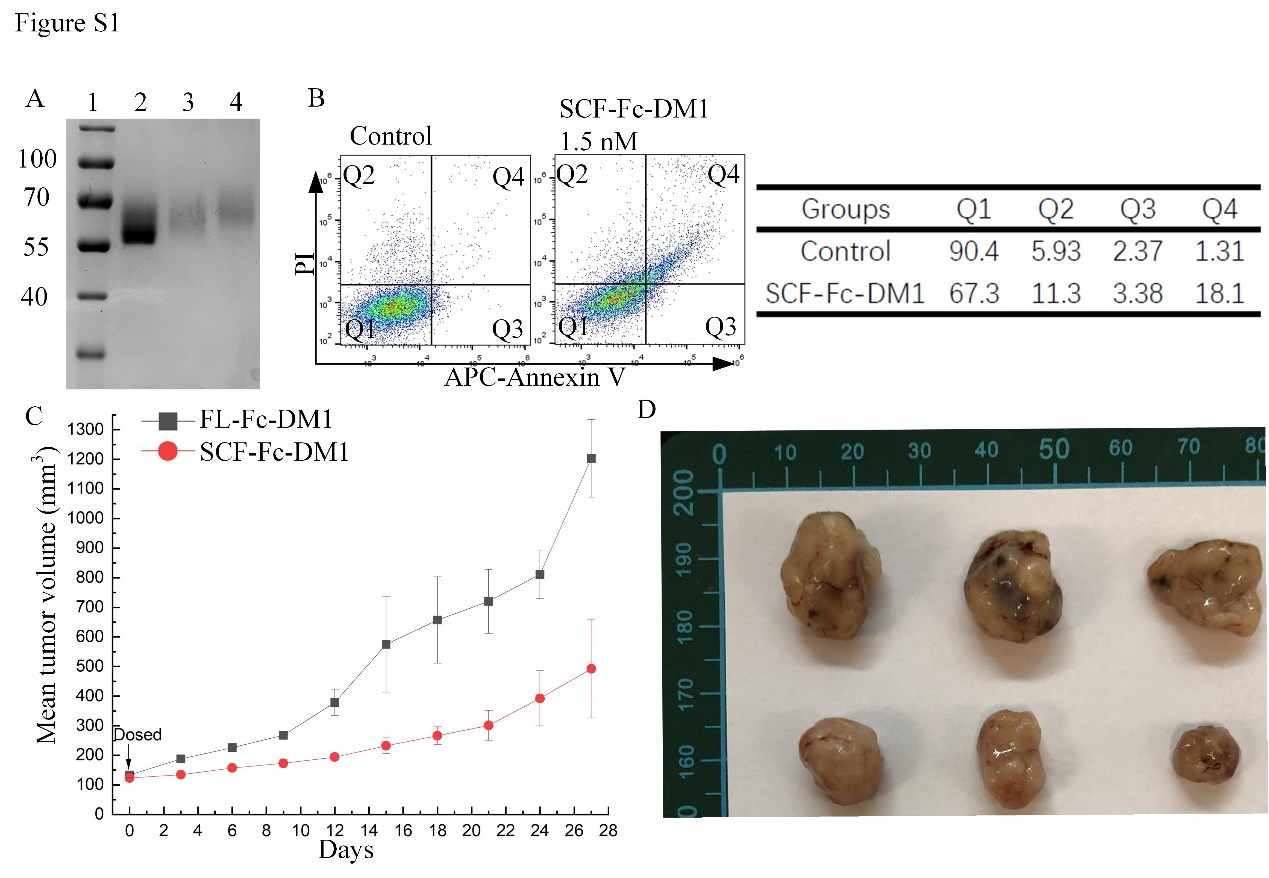


**Supplemental** **Figure Legends**

Supplemental Figure S1. The effect of SCF-Fc-DM1 on GIST 430 cells in mice. A. Conjugation of SCF-Fc and DM1. Lane1: pre-stained protein marker; Lane2: rhSCF-Fc protein; Lane3: rhSCF-Fc conjugated with SMCC (SCF-Fc-SMCC); Lane4: SCF-Fc-SMCC conjugated with DM1 (SCF-Fc-DM1). B. Effect of SCF-Fc-DM1 on GIST 430 cells *in vitro* by FACS analysis. C. Tumor volumes of mice treated with SCF-Fc-DM1 or FL-Fc-DM1 as the control. SCF-Fc-DM1 (6.0 mg/kg) and control (FL-Fc-DM1, 6.0 mg/kg) were administrated once on day zero. D. Tumors dissected from mice.
